# Imitation learning for improved 3D PET/MR attenuation correction^☆^

**DOI:** 10.1016/j.media.2021.102079

**Published:** 2021-07

**Authors:** Kerstin Kläser, Thomas Varsavsky, Pawel Markiewicz, Tom Vercauteren, Alexander Hammers, David Atkinson, Kris Thielemans, Brian Hutton, M.J. Cardoso, Sébastien Ourselin

**Affiliations:** a Department of Medical Physics & Biomedical Engineering, University College London, London WC1E 6BT, UK; b School of Biomedical Engineering & Imaging Sciences, King’s College London, London SE1 7EH, UK; c Kings College London & GSTT PET Centre, St. Thomas Hospital, London, UK; d Centre for Medical Imaging, University College London, London W1W 7TS, UK; e Institute of Nuclear Medicine, University College London, London NW1 2BU, UK

**Keywords:** MR to CT synthesis, Deep learning, Imitation learning, Convolutional neural network

## Abstract

The assessment of the quality of synthesised/pseudo Computed Tomography (pCT) images is commonly measured by an intensity-wise similarity between the ground truth CT and the pCT. However, when using the pCT as an attenuation map (μ-map) for PET reconstruction in Positron Emission Tomography Magnetic Resonance Imaging (PET/MRI) minimising the error between pCT and CT neglects the main objective of predicting a pCT that when used as μ-map reconstructs a pseudo PET (pPET) which is as similar as possible to the gold standard CT-derived PET reconstruction. This observation motivated us to propose a novel multi-hypothesis deep learning framework explicitly aimed at PET reconstruction application. A convolutional neural network (CNN) synthesises pCTs by minimising a combination of the pixel-wise error between pCT and CT and a novel metric-loss that itself is defined by a CNN and aims to minimise consequent PET residuals. Training is performed on a database of twenty 3D MR/CT/PET brain image pairs. Quantitative results on a fully independent dataset of twenty-three 3D MR/CT/PET image pairs show that the network is able to synthesise more accurate pCTs. The Mean Absolute Error on the pCT (110.98 HU ± 19.22 HU) compared to a baseline CNN (172.12 HU ± 19.61 HU) and a multi-atlas propagation approach (153.40 HU ± 18.68 HU), and subsequently lead to a significant improvement in the PET reconstruction error (4.74% ± 1.52% compared to baseline 13.72% ± 2.48% and multi-atlas propagation 6.68% ± 2.06%).

## Highlights

• CT-based losses neglect the PET data when doing MR to CT synthesis.
• MR to CT synthesis networks should account for PET reconstruction error.
• Multi-hypothesis uncertainty provides the best approximation of the true PET.
• Imitation learning results in a better optima and more generalisable model.

## Graphical abstract

**Figure:**
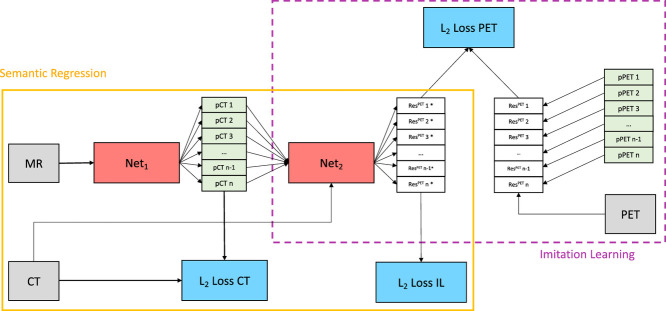

## 1 Introduction

The combination of Positron Emission Tomography (PET) and Magnetic Resonance Imaging (MRI) marked a significant event in the field of medical imaging, making it possible to simultaneously examine structural and functional characteristics of different tissue classes (Pichler et al., 2008) opening a way for many promising clinical applications (Torigian, Zaidi, Kwee, Saboury, Udupa, Cho, Alavi, 2013, Mader, Fuchs, Ferraro, Burger, 2020). In order to perform accurate regional quantification, it is essential to correct for photon attenuation of the whole imaging object (part of human body), i.e. the surrounding hardware (patient bed and supplementary coils) included, during the PET reconstruction. In standalone PET scanners this information is usually obtained from a transmission scan (Meikle et al., 1995).

In hybrid imaging systems that combine PET with Computed Tomography (CT), it is possible to determine the tissue attenuation coefficients (μ) directly from the CT image as Hounsfield units (HU) by using a bi-linear scaling method (Burger et al., 2002). However, such direct mapping is particularly challenging in PET/MRI due to the missing correlation between MR image intensities that are related to proton density and attenuation coefficients as opposed to the case when a CT image is available. While CT remains the clinically accepted gold-standard for PET/MR attenuation correction, it is desirable to generate accurate μ-maps without the need of an additional CT acquisition. Hence, the concept of synthesising pseudo CT (pCT) images from MRs has gained a lot of attention in the field of PET/MR attenuation correction (MRAC). Hofmann et al. pioneered in this field by combining pattern recognition and atlas registration methods and were the first to introduce the synonym pCT (Hofmann et al., 2008). They later showed that the combination of atlas registration and pattern recognition resulted in better PET quantification compared to segmentation based MRAC approaches (Hofmann et al., 2011). The majority of PET/MR scanners currently employ segmentation-based MRAC methods where pre-defined attenuation coefficients are assigned to different tissue classes (Berker, Franke, Salomon, Palmowski, Donker, Temur, Mottaghy, Kuhl, Izquierdo-Garcia, Fayad, et al., 2012, Yang, Wiesinger, Kaushik, Shanbhag, Hope, Larson, Seo, 2017). However, this can be particularly challenging due to the difficulty of separating bone from air. Thus, one field of MRAC focuses on the development of special MR sequences that use ultra short echo time (UTE) or zero echo time (ZTE) (Roy, Wang, Carass, Prince, Butman, Pham, 2014, Roy, Butman, Pham, 2017, Delso, Fernandez, Wiesinger, Jian, Bobb, Jansen, 2017, Delso, Gillett, Bashari, Matys, Mendichovszky, Gurnell, 2018, Su, Friel, Kuo, Al Helo, Baydoun, Stehning, Crisan, Traughber, Devaraj, Jordan, et al., 2019).

Recently, a multi-centre study (Ladefoged et al., 2017) has shown that multi-atlas propagation methods (Burgos et al., 2014) outperform methods that exploit emission data (Salomon, Goedicke, Schweizer, Aach, Schulz, 2010, Rezaei, Defrise, Bal, Michel, Conti, Watson, Nuyts, 2012) or use assigned tissue classes (Martinez-Möller, Souvatzoglou, Delso, Bundschuh, Chefd’hotel, Ziegler, Navab, Schwaiger, Nekolla, 2009, Catana, van der Kouwe, Benner, Michel, Hamm, Fenchel, Fischl, Rosen, Schmand, Sorensen, 2010) in order to correct for photon attenuation. Multi-atlas approaches estimate tissue density maps on a continuous scale by deforming an anatomical atlas that consists of paired MR and CT images to match the subject’s anatomy by using non-rigid registration.

Since then, there has been a shift of emphasis in the field of PET/MR attenuation correction towards deep learning approaches that have proved to be a powerful tool in the MR to CT image translation task, outperforming state-of-the-art multi-atlas-based methods (Burgos et al., 2014). Many deep learning methods employ convolutional neural networks (CNN) that are able to capture the contextual information between two image domains (as between MR and CT) in order to translate one possible representation of an image into another. Supervised learning settings require a training dataset that comprises sets of input images (i.e. MR) along with their corresponding target images (i.e. CT). In 2017, Han presented a 2D Deep CNN that directly learns a mapping function to translate a 2D MR image slice into its corresponding 2D CT image slice (Han, 2017) closely following the U-Net architecture, which has gained recognition in the deep learning community due to its strong performance in the field of image segmentation (Ronneberger et al., 2015). More recently, Kläser et al. presented a fully 3D deep CNN that generates pCT images recursively introducing a corrective network with shared parameters and deep supervision loss that reduces the residuals of an initial pCT prediction (Kläser et al., 2018).

**Fig. 1:**
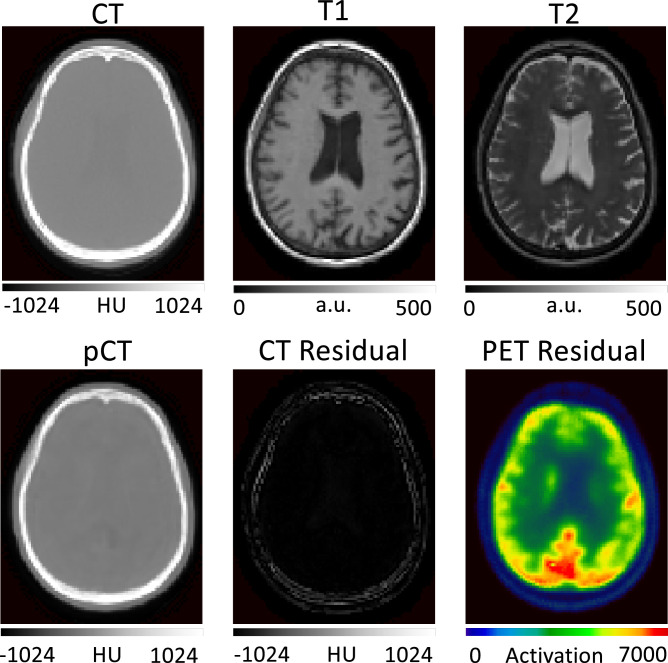
Top row: Ground truth CT, T1-weighted MRI, T2-weighted MRI. Bottom row: pseudo CT, absolute error between ground-truth and pseudo CT, and absolute error between PETs reconstructed using the ground-truth CT and pseudo CT for attenuation correction. Small and very localised difference in the CT lead to large errors in the PET image. We argue that algorithms should be optimising for PET residuals and not only for CT residuals when used for PET attenuation correction.

These previous works make use of a popular method to optimise image translation networks by minimising the error between the predicted pCT and the corresponding ground-truth CT, which is equivalent to minimising the $L_{2}$-loss. $L_{2}$-losses are a sensible loss metric when the optimal pCT for PET reconstruction is the one that is in terms of intensity the closest to the target ground truth CT. However, $L_{2}$-losses do not recognise that the main aim of CT synthesis is to generate a synthetic CT that, when used as attenuation map for PET reconstruction, makes the reconstructed PET as close as possible to the gold standard PET reconstructed with the true CT. Also, due to their risk-minimising nature $L_{2}$-losses ignore that small local differences between the predicted pCT and the ground-truth CT can significantly impact the reconstructed PET. An illustration of this downstream impact in PET reconstruction can be seen in Fig. 1.

With the emergence of the cycleGAN in 2017 (Zhu et al., 2017), a lot of work has been done in the field of unsupervised pCT synthesis. Unsupervised learning scenarios disregard the need of paired data and the $L_{2}$-loss. Wolterink et al. (2017) presented a CNN in their work that minimises an adversarial loss to learn a mapping fuction between MR and CT. This adversarial loss encourages the pCT to be indistinguishable from the ground-truth CT. An additional CNN aims to assure that the pCT corresponds to the actual input MR image. However, using a cycleGAN alone for pCT synthesis does not automatically ensure that pCT and ground-truth CT are structurally consistent. That means, that the reconstructed MR image is almost identical to the input MR, however, the pCT is significantly different from the ground-truth CT. Therefore Yang et al. (2020) proposed a cycleGAN containing structural constrains by minimising an additional structural consistency loss. In 2019, Jin et al. (2019) presented a method that tries to overcome the lack of structural consistency of the cycleGAN by combining paired and unpaired data in order to reduce the errors introduced during the registration process of paired data. Hiasa et al. adapt the original cycleGAN approach to include a gradient consistency loss particularly aiming at improving the accuracy at boundaries (Hiasa et al., 2018). The original cycleGAN was used on 2D images only, neglecting the axial spatial context. Gong et al. extended the framework to work fully in 3D (Gong et al., 2020). Tao et al. propose to use a generative network that further contains residual blocks and is trained in a conditional setting to assist the MR-based AC process (Tao et al., 2020). However, none of these methods incorporated the information available in PET images.

In contrast to synthesising pCT images as an interim step to correct for photon attenuation in PET images, attempts have been made to directly synthesise PET images from CT images. Bi et al. (2017) proposed an approach that produces pseudo PET (pPET) images by using a multi-channel generative adversarial network. Their model utilises information from both manually annotated labels and CT images in order to directly synthesise pPETs with high uptake regions. These regions are constrained by anatomical information derived by the CT. In 2018, Ben-Cohen et al. (2019) presented a model that combines a fully convolution network (FCN) and a conditional GAN that refines the pPET generated by the FCN. Unlike previous methods, this method is fully automated and does not require manual annotation to synthesise PET images. However, thus far direct PET synthesis has only been performed from CT images with the aim of lesion or tumor detection. Sikka et al. adapt the original 3D U-Net architecture to a global and non-linear cross-modal approach that estimates PETs from MR images directly (Sikka et al., 2018). Hwang et al. combine the traditional maximum-likelihood reconstruction of activity and attenuation (MLAA) method (Rezaei et al., 2012) with deep learning in order to overcome the limitations of MLAA (Hwang et al., 2018). Yaakub et al. propose a method to synthesise pseudo-normal PET images from MR images in a generative manner in order to identify regions of hypometabolism in PET images of epilepsy patients (Yaakub et al., 2019). The approach presented by Dong et al. learns a pCT from a non-attenuation corrected PET image that is then used for PET/MR AC (Dong et al., 2019). Arabi et al. attempt to perform direct attenuation and scatter correction in image space on four different PET radiotracers. Their method attempts to imitate the PET reconstruction process by learning the relation between PET images attenuation corrected with CT-derived AC maps and non-attenuation corrected PET images, thus not requiring anatomical images (Arabi et al., 2020, Arabi and Zaidi, 2020).

Other recent works such as (Chartsias, Joyce, Papanastasiou, Semple, Williams, Newby, Dharmakumar, Tsaftaris, 2018, Chartsias, Joyce, Papanastasiou, Semple, Williams, Newby, Dharmakumar, Tsaftaris, 2019) attempt to factorize images into spatial anatomical and non-spatial representations. They demonstrate that their method can not only be used to translate images but also for other medical image analysis tasks such as segmentation and regression. Joyce et al. also used a factorized representation learning setting, which does not rely on labeled images anymore (Joyce and Kozerke, 2019).

To the best of our knowledge, all CT synthesis methods concentrate on minimising the residuals of the predicted pCT. However, pCT synthesis only represents an interim stage when intended to correct for photon attenuation in PET/MR and thus creating an additional space for likely introduced errors. The aim of this work is to directly minimise the PET residuals. This is achieved by introducing a novel MR to CT synthesis framework that is composed of two separate CNNs. The first CNN synthesises multiple valid CT predictions using Multi-Hypothesis Learning instead of a single pCT only (Rupprecht et al., 2017). An oracle determines the predictor that generates the most correct pCT and only updates the weights with regards to the winning mode. This enables the first CNN to specialise in predicting pCTs with distinct features (e.g. skull thickness, bone density). A second CNN then uses imitation learning in order to predict the residuals between ground-truth PETs and PETs reconstructed with each valid pCT. In this setting, the second CNN acts as a metric that predicts the pPET residuals. By minimising this metric loss, the network learns to synthesise pCTs that will ultimately result in pPETs with lower residual error.

This paper is an extension of our preliminary work (Kläser et al., 2019). We extend the framework to work in 3D, perform a five-fold cross-validation and add an additional validation on a completely independent dataset.

## 2 Methods

### 2.1 Multi-hypothesis learning

Given a set of input MR images x∈X and a set of output CT images y∈Y, the proposed image synthesis approach aims to find a mapping function $f_{\phi }$ between the two image domains X and Y

(1) $$f_{\phi }:X\to Y\;\text{with}\;\phi \in R^{n}.$$

In a supervised learning scenario with a set of N paired training tuples $(x_{i},y_{i}),$ i=1,…,N, we try to find the predictor $f_{\phi }$ that minimises the error

(2) $$\frac{1}{N}\sum_{i=1}^{N}L\left(f_{\phi }\left(x_{i}\right),y_{i}\right).$$

L can be any desired loss, such as the classical $L_{2}$-loss. In the proposed multi-hypothesis scenario, the network provides multiple predictions of valid pCT realisations:

(3) $$f_{\phi }^{j}\left(x\right)\in \left(f_{\phi }^{1}\left(x\right),\ldots ,f_{\phi }^{M}\left(x\right)\right)\;\text{with}\;M\in N.$$

As in the original work for multi-hypothesis learning (Rupprecht et al., 2017), only the loss of the best predictor $f_{\phi }^{j}\left(x\right)$ will be used during training following a Winner-Takes-All (WTA) strategy, i.e.

(4) $$L\left(f_{\phi }\left(x_{i}\right),y_{i}\right)=min_{j\in [1,M]}L\left(f_{\phi }^{j}\left(x_{i}\right),y_{i}\right).$$

This way the network learns M modes to generate pCTs, where each mode specialises on specific features.

### 2.2 Imitation learning

Following the hypothesis that the $L_{2}$-loss is not optimal as a loss metric for pCT synthesis when used to correct for attenuation in PET/MR because of its risk minimising nature (e.g., the sinus region which is dark in T1-weighted MRI can be mapped to air or to bone but not to any value in between Cardoso et al., 2015), we propose to train a second CNN aiming to minimise subsequent PET residuals. This network approximates the function

(5) $$g_{\psi }:Y,\tilde{Y}\to Z\;\text{with}\;\psi \in R^{n},$$

by taking ground truth CTs ($y_{i}$) and pCTs ($f_{\phi }^{j}\left(x_{i}\right)\in \tilde{Y}$) as inputs. Here, Z is a set of error maps between the ground truth PET and the pPET that was reconstructed (as in Section 3.4) with each of the M pCT realisations as μ-maps. In other words, this second CNN learns to predict the PET reconstruction error from an input CT-pCT pair, thus imitating, or approximating, the PET reconstruction process. This imitation CNN is trained by minimising the $L_{2}$-loss between the true PET uptake error z and the predicted error $\tilde{z},$ i.e.

(6) $$L_{2}={∥z-\tilde{z}∥}_{2}.$$

Lastly, we use this second CNN as a new loss function for the first CNN and minimise the Root Mean Squared Error (RMSE), as it provides an approximate and differentiable estimate of the PET residual loss. Thus, the loss minimised by the first CNN is defined as

(7) $$\begin{array}{cc} L(f_{\phi }\left(x_{i}\right),y_{i},z_{i})= & \;\min_{j\in [1,M]}L\left(f_{\phi }^{j}\left(x_{i}\right),y_{i}\right)\\ & +\min_{j\in [1,M]}\left[g_{\psi }\left(f_{\phi }^{j}\left(x_{i}\right),y_{i}\right),z_{i}\right]. \end{array}$$

### 2.3 Proposed network architecture

**Fig. 2:**
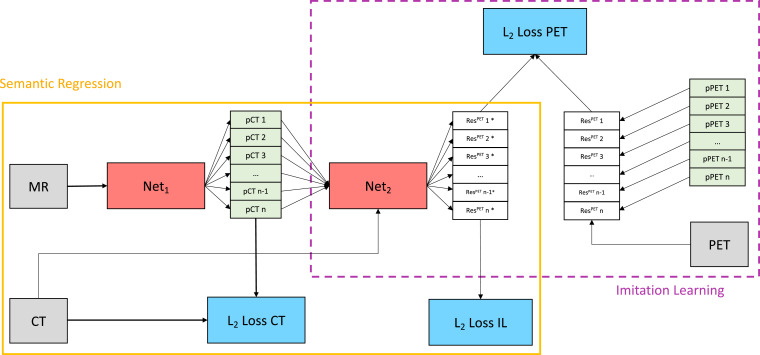
Yellow solid box: semantic regression. A first CNN ($Net_{1}$) with MR images as inputs predicts multiple valid pCT realisations by minimising a combination of the $L_{2}$-loss between true CT and pCT ($L_{2}$-loss CT) and a learned metric loss ($L_{2}$-loss IL). In the first training stage only $L_{2}$-loss CT is considered and $L_{2}$-loss IL is weighted to zero. Purple dashed box: imitation network. A second CNN ($Net_{2}$) with pCTs and corresponding CTs as input predicts the residuals between PET reconstructed with true CT-derived μ-map and pPET reconstructed with pCT as μ-map by minimising $L_{2}$-loss PET. The training of semantic regression and imitation network is performed in three separate stages. (For interpretation of the references to colour in this figure legend, the reader is referred to the web version of this article.)

The proposed network architecture presented in Fig. 2 is trained in three distinct phases. In the first stage, a HighRes3DNet (Li et al., 2017) with multiple hypothesis outputs is trained minimising an $L_{2}$-WTA loss in order to generate different pCT realisations (Fig. 2 yellow solid box). In the second stage, the weights of the first network are frozen and a second instance (Fig. 2 purple dashed box) of HighRes3DNet is trained. This second network learns to predict the residual between the PET reconstructed with the true CT-derived μ-map and the pPET that was reconstructed using the μ-map derived from each pCT to correct for attenuation. This way the network learns the mapping between pCT residual and subsequent pPET reconstruction error. Note, that the network was trained on reconstructed PET images (see Section 3.4). In the final stage, the first network is retrained with a combination of the CT $L_{2}$-loss and the proposed metric loss in equal proportions. Thus the network minimises both the CT residual and the pPET reconstruction error.

### 2.4 Implementation details

During the training stage subvolumes of size 56×56×56 pixels were randomly sampled from the input data (T1- and T2-weighted multi-channel input) due to a limited GPU memory budget. Those patches were augmented by randomly rotating each of the three orthogonal planes on the fly by an angle in the interval of [10${}^{\circ },$ 10${}^{\circ }$]. Further augmentations on the MR data included random scaling by a factor between 0.9 and 1.1, random bias field augmentation of all three planes and random noise in a range between 10 SNR and 25 SNR. We performed a five-fold cross-validation, where for each fold the data were split into 70% training, 10% validation and 20% testing data. All training phases were performed on a Titan V GPU with Adam optimiser (Kingma and Ba, 2014). Within the first training stage a model was trained for 50k iterations with a learning rate of 0.001. The network of the second training stage learning to minimise the pPET reconstruction error was trained for 500k iterations with a learning rate of 0.001. Within the final training stage a complete model was trained for 100k iterations minimising a combination of the proposed losses with a learning rate of 0.001 before decreasing the learning rate by a factor of 10 and resuming training until convergence. The framework was implemented in NiftyNet, an open-source TensorFlow-based CNN platform for research in the medical image analysis domain (Gibson et al., 2017).

## 3 Experimental datasets and materials

### 3.1 Training dataset

The experimental dataset used for training and cross-validation consisted of twenty pairs of brain MR, CT and ${}^{18}$F-FDG PET images. All 20 subjects were scanned on a 3T Siemens Magneton Trio scanner and T1-weighted (3.0 T; TE/TR/TI,2.9 ms/2200 ms/900 ms; flip angle 10${}^{\circ }$; voxel size 1.1×1.1×1.1 mm${}^{3}$) and T2-weighted (3.0 T; TE/TR, 401 ms/3200 ms; flip angle 120${}^{\circ }$; voxel size 1.1×1.1×1.1 mm${}^{3}$) volumetric scans were acquired. PET/CT imaging was performed on a GE Discovery ST PET/CT scanner providing CT images (voxel size 0.586×0.586×2.5 mm${}^{3},$ 120 kVp, 300 mA) and reconstructed PET images (voxel size 1.95×1.95×3.27 mm${}^{3}$).

### 3.2 Independent validation dataset

In order to validate the proposed method on a completely independent dataset twenty-three subjects were scanned on a GE Discovery 710 PET/CT scanner providing CT images (voxel size 1.367×1.367×3.27 mm${}^{3},$ 140 kVp, 10 mA) and reconstructed ${}^{18}$F-FDG PET images (1.0×1.0×3.27 mm${}^{3}$). The 23 subjects were then scanned on a Siemens Biograph mMR PET/MR immediately after. T1-weighted images were acquired using a three-dimensional magnetisation-prepared rapid gradient-echo (MP RAGE) sequence (Brant-Zawadzki et al., 1992) (3.0 T; TE/TR/TI,2.63 ms/1700 ms/900 ms; flip angle 9${}^{\circ }$; voxel size 1.1×1.1×1.1 mm${}^{3}$). Three-dimensional isotropic T2-weighted images were acquired with a fast/turbo spin-echo sequence (SPACE) (3.0 T; TE/TR, 383 ms/2700 ms; flip angle 120${}^{\circ }$; voxel size 1.3×1.3×1.3 mm${}^{3}$).

### 3.3 Database processing

For each subject in the training database, MRs and CTs were affinely aligned using a symmetric approach (Modat et al., 2014) based on Ourselin et al. (2001) followed by a fully affine registration in order to compensate for possible gradient drift in the MR images. We then performed a very low degree of freedom non-rigid deformation in order to compensate for different neck positioning before implementing a second non-linear registration, using a cubic B-spline with normalised mutual information (Modat et al., 2010). For the purpose of this work, the data were resampled to the original Siemens Biograph mMR PET resolution of 344×344×127 voxels with a voxel size of approximately 2×2×2 mm${}^{3}$. We extracted two masks for evaluation purposes, a head mask from the CT and a brain mask from the T1-weighted MR image. The head mask was generated by thresholding the CT at 500 HU thus excluding the background from the performance metric analysis. The additional brain mask was extracted from the T1-weighted MR image to exploit the radionuclide uptake in the brain region only. We reconstructed three PETs with each of the multi-hypothesis pCTs (here denoted as pPET) in order to train the imitation CNN, resulting in a total of 60 pCT/pPET pairs. CT and MR images within both datasets were linearly rescaled to be between 0 and 1 as it has been found to increase stability during training. PET images were used in their original intensity range.

### 3.4 PET reconstruction

**Fig. 3:**
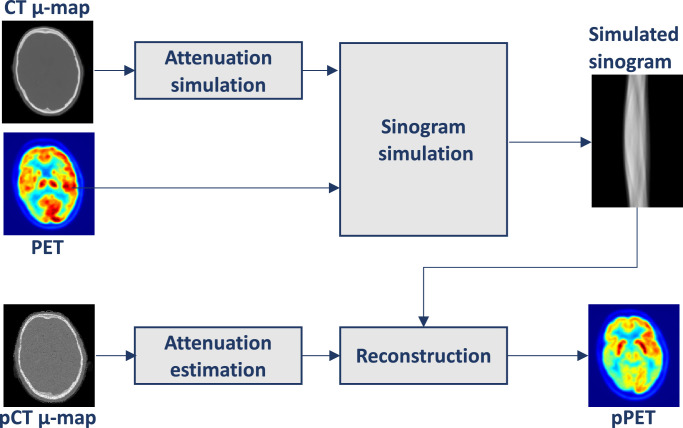
PET simulation: a PET forward projection is applied on the μ-map transformed CT to obtain attenuation factor sinograms. Similar forward projection is applied to the original PET to obtain simulated emission sinograms. Final pPETs are reconstructed from simulated emission sinograms using pCT derived attenuation maps.

3D PET images were reconstructed using NiftyPET, an open-source package for high-throughput PET image reconstruction (Markiewicz et al., 2018). We did not have access to the raw PET data and therefore the following simulation was performed (see Fig. 3): attenuation factor sinograms were generated by forward projecting the μ-map transformed versions of each pCTs. Simulated emission sinograms were acquired using a similar forward projection applied to the original PET images. The simulated emission sinograms are then attenuated through element-wise multiplication with the attenuation factor sinograms. We then reconstructed the resulting sinograms with the original CT-based μ-map in order to obtain a reference image. In the same manner, reconstruction was performed using the μ-maps derived from each pCT.

## 4 Validation and results

**Fig. 4:**
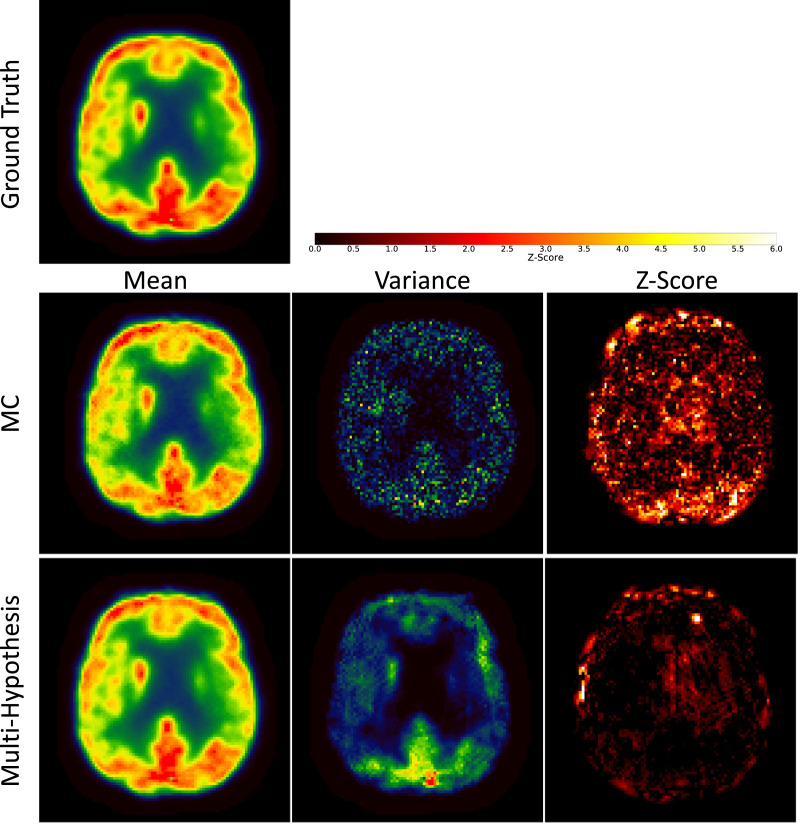
PET values (first column), variance (middle column) and *Z*-score (right column) of ground truth PET (top row) compared to pPET values reconstructed with pCTs from Monte Carlo (MC) dropout sampling (middle row) and pCTs from multi-hypothesis sampling (bottom row). The multi-hypothesis model captures true PET values better than MC dropout method.

In a first experiment, we evaluated the use of two sampling schemes to synthesise multiple pCT realisations: test-time Monte-Carlo (MC) dropout (Gal and Ghahramani, 2016) versus multi-hypothesis learning. The results are demonstrated in Fig. 4. The intensities of pPETs reconstructed with a μ-map from the pCTs generated with MC dropout show an artificially low variance, whereas the intensities of pPETs reconstructed with the pCTs synthesised with the proposed multi-hypothesis model provide a wider distribution. In order to investigate the accuracy of the predictions, we calculated the *Z*-score of the ground-truth PET with regards to each sampling scheme to demonstrate the relationship between the mean data distribution and the ground truth PET. Fig. 4-Right presents the per pixel *Z*-score defined as

(8) $$\frac{\text{PET}-\mu ({\text{pPET}}^{M})}{\sigma ({\text{pPET}}^{M})},$$

where $\mu ({\text{pPET}}^{M})$ and $\sigma ({\text{pPET}}^{M})$ are the per-pixel average and per pixel variance over M pPET samples respectively. Results show that a significantly lower *Z*-score can be found in the brain region for the multi-hypothesis model compared to when MC dropout is used. This means that the multi-hypothesis-based PET uncertainty does encompass the true PET intensity more often than the competing MC dropout method.

**Fig. 5:**
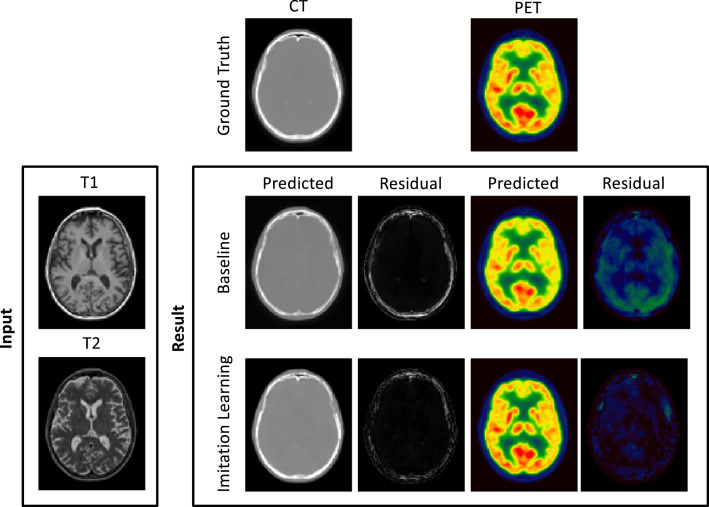
Qualitative results. From top to bottom: Ground-truth, baseline (HighRes3DNet) and imitation learning alongside input MR images. From left to right: CT, pCT-CT residuals, PET, pPET-PET residuals. The error in the pCT generated with the proposed imitation learning is lower than the baseline pCT residuals. The error in the pPET reconstructed with the proposed method is significantly lower than the pPET error for the baseline method.

Following the results of the first experiment, we trained a fully 3D model on the first dataset (see Section 3.1) and performed a five-fold cross-validation. Qualitative results are presented in Fig. 5. The first column shows the true CT image (top), a pCT synthesised with the HighRes3DNet chosen as baseline method (middle) and a pCT synthesised using the proposed imitation learning (bottom). Next to the CTs (2nd column) the corresponding residuals between pCT and true CT are illustrated. In the third column the ground truth PET (top), baseline pPET (middle) and the imitation learning pPET (bottom) are shown followed by the resulting pPET residuals in the last column. For quantification purposes, we calculated the Mean Absolute Error (MAE) defined as

(9) $$MAE=\frac{\sum |pCT-CT|}{V}$$

of the pCTs only in the number of voxels in a region of interest (V), here head and brain only region. We validated the advantages of the proposed imitation learning model on the remaining 20% of the dataset hold out for testing (see Table 1). The proposed method leads to a lower MAE on the CT (79.04 HU ± 3.57 HU) compared to the simple feed forward model (92.77 HU ± 8.57 HU), the Mean Absolute Percentage Error (MAPE) in the resulting pPET is significantly lower (paired t-test, p<0.05) for the proposed method (4.04% ± 0.50% for brain region; 5.62% ± 0.21% for whole head) when compared to the baseline model (5.60% ± 1.25% for brain region; 7.26% ± 0.92% for whole head).

**Table 1:** Mean Absolute Error (MAE) in pCT generated with HighRes3DNet and imitation learning pCTs and corresponding MAE in pPET in the brain region only and in the whole head for all five folds.

Fold	MAE CT (in HU)		MAPE PET brain (in %)		MAPE PET head (in %)	
	Baseline	Imitation learning	Baseline	Imitation learning	Baseline	Imitation learning
1	103.93±14.46	79.97±10.19	7.45±1.55	4.11±0.71	8.62±1.88	5.51±0.80
2	99.70±15.65	82.32±7.91	6.24±0.94	4.70±1.28	7.74±1.24	5.59±0.86
3	89.17±10.49	81.44±7.49	4.60±1.51	3.63±0.77	6.62±1.42	5.91±0.55
4	86.71±10.99	78.17±0.22	5.21±1.04	3.48±0.33	6.91±0.96	5.72±0.62
5	84.33±7.19	73.30±2.26	4.48±0.97	4.28±0.63	6.40±0.88	5.37±0.56
Average	92.77±8.57	79.04±3.57	5.60±1.25	4.04±0.50	7.26±0.92	5.62±0.21

In order to validate the previously trained fully 3D model on a completely independent dataset, the performance of the proposed method was compared against ground truth data of 23 subjects. The method was further compared to the chosen baseline method (HighRes3DNet only) and a multi-atlas propagation method that is routinely used in clinical practice and clinical trial setting (Burgos et al., 2014). The quantitative validation was performed in two steps:

1. Pseudo CTs were synthesised from all 23 subject’s MR images using the proposed method, the baseline method and the multi-atlas propagation approach. All generated pCTs were then compared to the subject’s ground truth CT to validate the accuracy of the synthesis.
2. Pseudo PET images were reconstructed following the simulation described in Section 3.4 using μ-maps generated with pCTs from proposed, baseline and multi-atlas method. All pPETs were then compared to the ground truth PET that was reconstructed using the μ-map extracted from the original CT in order to validate the accuracy of the PET attenuation correction.

Fig. 5 shows the ground truth CT and pCTs synthesised with the proposed imitation learning and the baseline model and the corresponding residuals as well as predicted pPET images and pPET residuals.

The results of the independent validation are shown in Table 2. The MAE over all 23 subjects in the CT for the proposed method is 110.98 HU ± 19.22 HU compared to the baseline 172.12 HU ± 19.61 HU and a multi-atlas propagation method 153.40 HU ± 18.68 HU. Subsequently, the average MAPE of all reconstructed PET images within the brain for the proposed method is around 3 times lower than the MAPE of the baseline (4.74% ± 1.52% compared to 13.72% ± 2.48%) in the brain region and 2.4 times lower in the whole head region (9.05% ± 1.93% compared to 21.51% ± 3.14%). Further, the proposed imitation learning method achieves an approximately 1.4 times lower average MAPE of all reconstructed PET images in both the head and the brain region compared to PET images reconstructed with the pCT generated with the multi-atlas propagation method (6.68% ± 2.06% in brain region, 12.00% ± 2.11% in head region).

**Table 2:** Mean Absolute Error (MAE) in pCT generated with HighRes3DNet, multi-atlas propagation and imitation learning pCTs and corresponding MAE in pPET in the brain and head region only on independent dataset.

MAE CT (in HU)			MAPE PET brain (in %)			MAPE PET head (in %)		
Baseline	Multi-atlas	Imitation learning	Baseline	Multi-atlas	Imitation learning	Baseline	Multi-atlas	Imitation learning
172.12±19.61	153.40±18.68	110.98±19.22	13.72±2.48	6.68±2.06	4.74±1.52	21.51±3.14	12.00±2.11	9.05±1.93

**Fig. 6:**
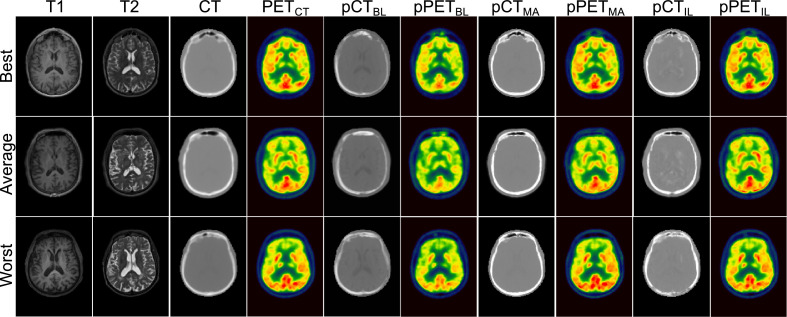
From left to right: the acquired T1-, T2-weighted MRI, CT, and ground truth ${}^{18}$F-FDG PET, the pCT and pPET generated with the baseline (HighRes3DNet only), the pCT and pPET generated with the multi-atlas propagation, and the pCT, and pPET generated with the proposed imitation learning for the subjects within the independent validation dataset that obtained the lowest (top row), average (middle row), and highest (bottom row) MAPE in the pPET, which was consistent among all methods.

Example images of T1-, T2-weighted, CT, pCT synthesised with baseline method, multi-atlas propagated pCT and pCT generated with proposed method and corresponding reconstructed PET images are presented in Fig. 6 for three subjects whose pPET showed the lowest, the average, and the highest MAPE.

**Fig. 7:**
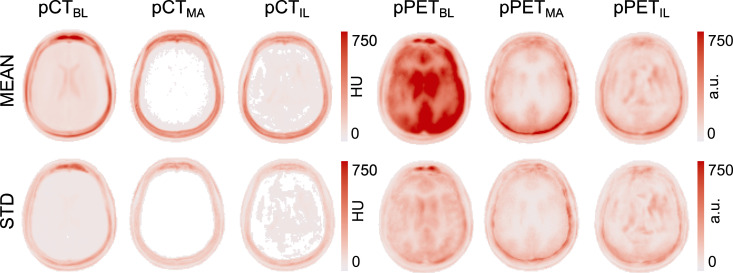
Groupwise average over 23 subjects (top) and standard deviation (bottom) of the pCT absolute residuals (in HU) of baseline, multi-atlas propagation and imitation learning (column 1–3) and pPET absolute residuals (in arbitrary unit (a.u.)) between gold-standard PET and pPETs reconstructed with baseline pCT, multi-atlas propagation pCT and imitation learning pCT (column 4–6).

Lastly, both the pCT images and the pPET images were mapped to a common space following a CT-based groupwise registration method (Rohlfing et al., 2001). This groupwise registration method is a repeated application of an intensity-based non-rigid registration algorithm based on third-order 3D B-splines. It was introduced to generate an average atlas from 3D images. We performed five affine registration loops followed by ten non-rigid registration loops with B-spline spacing of five voxels. This registration was not directly performed on the pCT residuals rather on the original CT images. The voxelwise transformation was then applied to the pCT residuals in order to propagate them into a common space. We then computed the average across all subjects of the absolute pCT error map and the absolute pPET error map (Fig. 7 top). We note that the average error in the pCT for all three methods is centered in the skull region and only shows small improvement for the pCT generated with the proposed imitation learning. However, looking at the absolute difference of the pPET and the gold standard PET, we note that the average uptake error in the pPET reconstructed with the baseline pCT is significantly higher than in the pPET reconstructed with the pCT synthesised with the proposed imitation learning. Further, we observe that small intensity differences in the skull region in the pCT generated with the multi-atlas propagation method cause a significantly higher uptake error in the pPET when this pCT is used for pPET reconstruction. The bottom row of Fig. 7 shows the standard deviation across all 23 subjects of pCT and pPET difference maps. We observe that the standard deviation in the average pCT error map is smaller for the proposed method compared to the baseline and the multi-atlas propagation method. Furthermore, the standard deviation of the groupwise average pPET error is significantly higher for the pPET difference map that was computed between the pPET reconstructed with the baseline method and the gold standard PET compared to the pPET difference map that was generated between the pPET reconstructed with the proposed imitation learning method and the gold standard PET.

## 5 Discussion

Following the hypothesis that the classical $L_{2}$-loss is not necessarily the optimal minimisation metric for CT synthesis, the presented multi-stage imitation learning framework minimises a combination (as in Eq. (7)) of the pixel-wise error between pCT and CT and a proposed metric-loss that itself is represented by a CNN explicitly aiming at PET reconstruction application.

Two separate datasets were used in this work; one for training and cross-validation and another completely independent dataset to evaluate the performance of the proposed method on input images that were acquired with a different imaging protocol. We compared the performance of our imitation learning framework to a feed forward network for pCT synthesis that minimises the classical $L_{2}$-loss. The results of the five-fold cross-validation in Table 1 demonstrate that the mean absolute error between the generated pCT and the acquired ground-truth CT is significantly lower compared to the baseline method for each fold. We hypothesise that this is likely due to the regularising nature of the imitation learning loss as all networks were trained until convergence. We further note that the standard deviation for the proposed method is generally lower than the standard deviation of the baseline method. The lower error in the pCT images subsequently results in a lower error in the reconstructed pPET image when the pCT is used as attenuation map for the PET reconstruction. The MAPE in the whole head region and in the brain region only is significantly lower for the pPET reconstructed with the proposed pCT compared to the baseline pCT. Difference images in Fig. 5 reveal that the errors in the pCT are concentrated in the skull area, especially in areas with air/bone and soft-tissue/bone boundaries like the nasal cavities. The wrongly predicted intensities in the skull region lead to wrong attenuation maps that in turn lead to an overall underestimation of radionuclide uptake in the reconstructed pPET images as shown in Fig. 5.

Quantitative results on a completely independent validation dataset are presented in Table 2 and confirm the improved performance of the proposed imitation learning network. We extended the validation on the independent dataset by an additional comparison to a multi-atlas propagation method from Burgos et al. (2014) that is robust to image domain shifts. Results show that the error of the proposed pCT lies around 111 HU whereas the baseline pCT error is around 172 HU, which shows an improvement of approximately 35%. Even though a smaller pCT error was not necessarily the aim of this work, the introduction of the imitation learning method has resulted in a better optima and more generalisable model. Comparing the performance of our novel deep learning framework exploiting a combined pixel-wise and metric loss to the multi-atlas propagation method that is routinely used in clinical practice and clinical trial settings, the proposed method improves the pCT synthesis performance by approximately 28%. The impact of the synthesis error in the pCT on the pPET is particularly present on the independent dataset that consisted of T1- and T2-weighted images that were acquired with a different imaging protocol than the training input MR data. The MAPE in the pPET reconstructed with the baseline is approximately 3 times higher and 1.4 times higher for the pPET reconstructed with the multi-atlas propagated pCT compared to the pPET reconstructed with the proposed imitation learning pCT (13.72% ± 2.48% compared to 6.68% ± 2.06% and 4.74% ± 1.52%). Qualitative results in Fig. 6 illustrate the pCTs and corresponding pPETs of the independent validation and emphasise the underestimation of the skull in the baseline method and its missing ability to generate air/bone boundaries properly whereas pCTs generated with the proposed method seem sharper than the ground-truth CT images leading to pPET images that reconstruct the radionuclide uptake more accurately. The pCTs generated with the multi-atlas propagation method look visually sharper than the pCT generated with the imitation learning method, however, the density of the bone is overestimated which leads to an inaccurately radionuclide uptake in the reconstructed pPET.

Analysing the groupwise average difference and standard deviation across all 23 subjects of the independent dataset shows a similar performance on the pCT synthesis for baseline, multi-atlas propagation and proposed imitation learning method as demonstrated in Fig. 7. However, when exploiting the average error map of the reconstructed radionuclide uptake the baseline method shows a significantly higher uptake error particularly in the brain region compared to the other two methods. The higher average difference in the skull region of the pCT generated with the multi-atlas propagation method leads to a higher average error in the resulting pPET image especially close to the skull. All three attenuation correction methods introduce a bias but the variance of the bias is lower when the pPET is corrected with the attenuation map derived from the imitation learning pCT.

The results of the validation on the independent dataset show a common problem of deep learning methods: image domain shift. Often methods are developed to serve a problem specific purpose making them less generalisable, i.e. testing on images that are from a slightly different domain (here different MR acquisition protocols) than the training data fails. Multi-atlas propagation methods are robust to this problem since they rely on structural similarities in the image rather than voxel-wise intensity similarities. The proposed method shows to have good extrapolation properties due to a more realistic metric, which leads to less domain shift issues and an improved performance. Structural similarities could be included in the proposed network in the future in order to mitigate the domain shift issue. This could for example be implemented as a structural similarity map applied to the loss function during training.

In this work we provide a proof of concept showing that minimising a combined loss that consists partly of the classical $L_{2}$-loss and partly of a learned metric loss that itself minimises the error in the reconstructed pPET when the pCT is used as attenuation map can indeed significantly improve the PET reconstruction accuracy compared to using an $L_{2}$-loss only. Effects in PET images are non-linearly related to the CT, which is compensated by the novel imitation loss. The proposed concept of a combined $L_{2}$-loss and learned metric loss can be applied to other networks such as U-Net or Deep Boosted Regression that have shown promising results in the MR to CT image translation task.

As a consequence of the newly introduced imitation learning loss, we were further able to improve the performance of the pCT synthesis on an image-based level when optimising our network not only for the pCT but also the pPET error. Since we are optimising over a high-dimensional model in a deep learning scenarios the introduction of the imitation loss appears to regularise the optimisation function landscape better.

However, supervised deep learning based methods for pCT synthesis for the purpose of MR attenuation correction also have limitations relying on a co-registered database that represents a wide range of the population’s anatomy. Small inaccuracies in the registration quality of the MR/CT database can have an influence on the training success. But, when validating on a database of images acquired with a different imaging protocol, the proposed method is robust to local registration inaccuracies and acquisition protocol changes generating pCT images that are significantly better than methods used in clinical practice. After all, accurately aligning CT and MR images is inevitable in order to validate the pixel-wise performance of any image synthesis algorithm until other appropriate methods have been developed that allow to validate on non-registered data.

Current limitations of the method are due to limited anatomical information in CT and MR images such as tumours as well as the tracer specificity of the proposed model. A larger database containing subjects with anatomical abnormalities could improve the robustness of the model. An uncertainty measure of the pCT prediction could be integrated in the network providing a means of safety checking in order to make the method robust for clinical use by declining predictions that are highly uncertain if any extreme abnormalities in the input MR image are present that could cause the model to fail.

The focus of this paper is on MR-derived attenuation correction for brain applications and requires further experiments to determine its suitability for other regions of the body. In theory, the proposed network could be applied to any body part assuming that the registration between MRI and CT is sufficiently accurate and morphological variability within the database is present.

## 6 Conclusion

In this work we proposed a novel deep learning framework for pCT synthesis in 3D for the purpose of PET/MR attenuation correction. Compared to state-of-the-art image synthesis CNNs, the proposed method does not assume the $L_{2}$-loss, that is commonly used as a minimisation metric in CT synthesis methods, as optimal when the ultimate aim is a low error in the corresponding pPET when used as μ-map. Quantitative analysis on an out-of distribution dataset shows that minimising a more suitable metric that indeed optimises for PET residuals (from CTs and pCTs) can improve the process of CT synthesis for PET/MR attenuation correction. We were further able to show that the proposed method is robust to changes in the imaging protocol of the input T1- and T2-weighted MR images. Overall the proposed method provides a significant improvement in PET reconstruction accuracy when compared to a simple feed forward network and a multi-atlas propagation approach.

## CRediT authorship contribution statement

**Kerstin Kläser:** Conceptualization, Methodology, Software, Visualization, Validation, Data curation, Writing - original draft. **Thomas Varsavsky:** Methodology, Software, Writing - review & editing. **Pawel Markiewicz:** Software, Writing - review & editing. **Tom Vercauteren:** Conceptualization, Writing - review & editing. **Alexander Hammers:** Data curation, Writing - review & editing. **David Atkinson:** Writing - review & editing. **Kris Thielemans:** Funding acquisition, Writing - review & editing. **Brian Hutton:** Writing - review & editing. **M.J. Cardoso:** Supervision, Conceptualization, Methodology, Writing - review & editing. **Sébastien Ourselin:** Supervision, Funding acquisition, Writing - review & editing.

## Declaration of Competing Interest

The authors declare that they have no known competing financial interests or personal relationships that could have appeared to influence the work reported in this paper.
